# Structural changes in Schwann cells and nerve fibres in type 1 diabetes: relationship with diabetic polyneuropathy

**DOI:** 10.1007/s00125-023-06009-z

**Published:** 2023-09-20

**Authors:** Xiaoli Hu, Christian Selmer Buhl, Marie Balle Sjogaard, Karoline Schousboe, Hatice Isik Mizrak, Huda Kufaishi, Troels Staehelin Jensen, Christian Stevns Hansen, Knud Bonnet Yderstræde, Ming-Dong Zhang, Patrik Ernfors, Jens Randel Nyengaard, Pall Karlsson

**Affiliations:** 1 Core Center for Molecular Morphology, Section for Stereology and Microscopy, Aarhus University, Aarhus, Denmark; 2 Steno Diabetes Center Aarhus, Aarhus University Hospital, Aarhus, Denmark; 3 Danish Pain Research Center, Department of Clinical Medicine, Aarhus University, Aarhus, Denmark; 4 Steno Diabetes Center Odense, Odense University Hospital, Odense, Denmark; 5 Steno Diabetes Center Copenhagen, Herlev, Denmark; 6 Department of Neurology, Aarhus University Hospital, Aarhus, Denmark; 7 Department of Medical Biochemistry and Biophysics, Division of Molecular Neurobiology, Karolinska Institutet, Stockholm, Sweden; 8 Department of Pathology, Aarhus University Hospital, Aarhus, Denmark

**Keywords:** Diabetic polyneuropathy, Neuropathic pain, Nociceptive Schwann cell, Skin punch biopsy, Type 1 diabetes

## Abstract

**Aims/hypothesis:**

Our aim was to investigate structural changes of cutaneous Schwann cells (SCs), including nociceptive Schwann cells (nSCs) and axons, in individuals with diabetic polyneuropathy. We also aimed to investigate the relationship between these changes and peripheral neuropathic symptoms in type 1 diabetes.

**Methods:**

Skin biopsies (3 mm) taken from carefully phenotyped participants with type 1 diabetes without polyneuropathy (T1D, *n*=25), type 1 diabetes with painless diabetic polyneuropathy (T1DPN, *n*=30) and type 1 diabetes with painful diabetic polyneuropathy (P-T1DPN, *n*=27), and from healthy control individuals (*n*=25) were immunostained with relevant antibodies to visualise SCs and nerve fibres. Stereological methods were used to quantify the expression of cutaneous SCs and nerve fibres.

**Results:**

There was a difference in the number density of nSCs not abutting to nerve fibres between the groups (*p*=0.004) but not in the number density of nSCs abutting to nerve fibres, nor in solitary or total subepidermal SC soma number density. The overall dermal SC expression (measured by dermal SC area fraction and subepidermal SC process density) and peripheral nerve fibre expression (measured by intraepidermal nerve fibre density, dermal nerve fibre area fraction and subepidermal nerve fibre density) differed between the groups (all *p*<0.05): significant differences were seen in participants with T1DPN and P-T1DPN compared with those without diabetic polyneuropathy (healthy control and T1D groups) (all *p*<0.05). No difference was found between participants in the T1DPN and P-T1DPN group, nor between participants in the T1D and healthy control group (all *p*>0.05). Correlational analysis showed that cutaneous SC processes and nerve fibres were highly associated, and they were weakly negatively correlated with different neuropathy measures.

**Conclusions/interpretation:**

Cutaneous SC processes and nerves, but not SC soma, are degenerated and interdependent in individuals with diabetic polyneuropathy. However, an increase in structurally damaged nSCs was seen in individuals with diabetic polyneuropathy. Furthermore, dermal SC processes and nerve fibres correlate weakly with clinical measures of neuropathy and may play a partial role in the pathophysiology of diabetic polyneuropathy in type 1 diabetes.

**Graphical Abstract:**

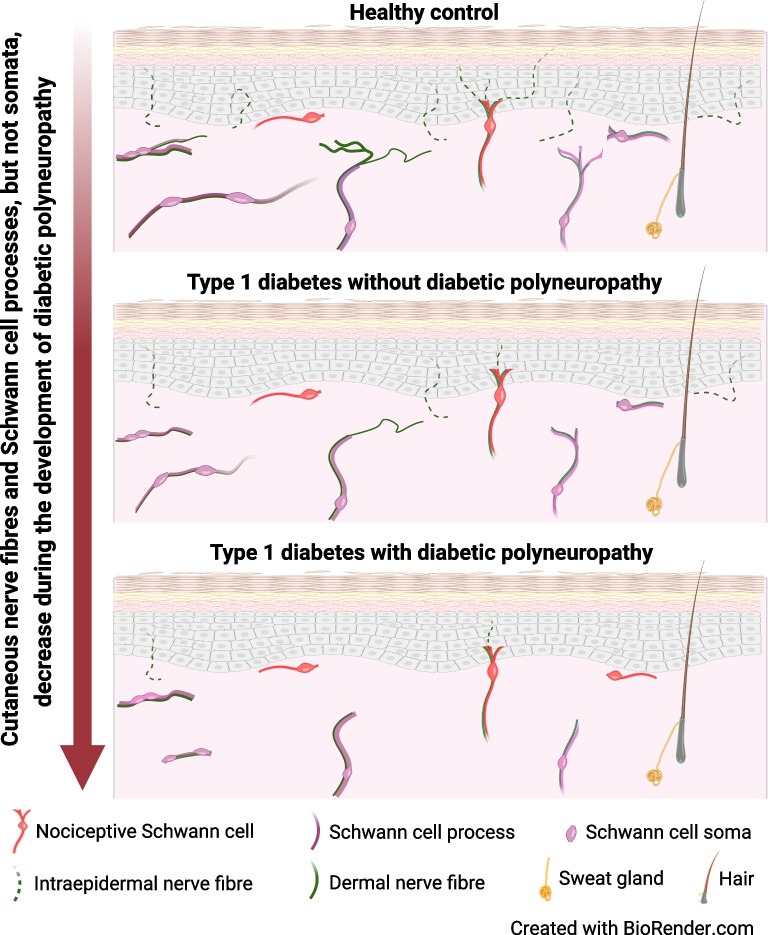

**Supplementary Information:**

The online version contains supplementary material available at 10.1007/s00125-023-06009-z.


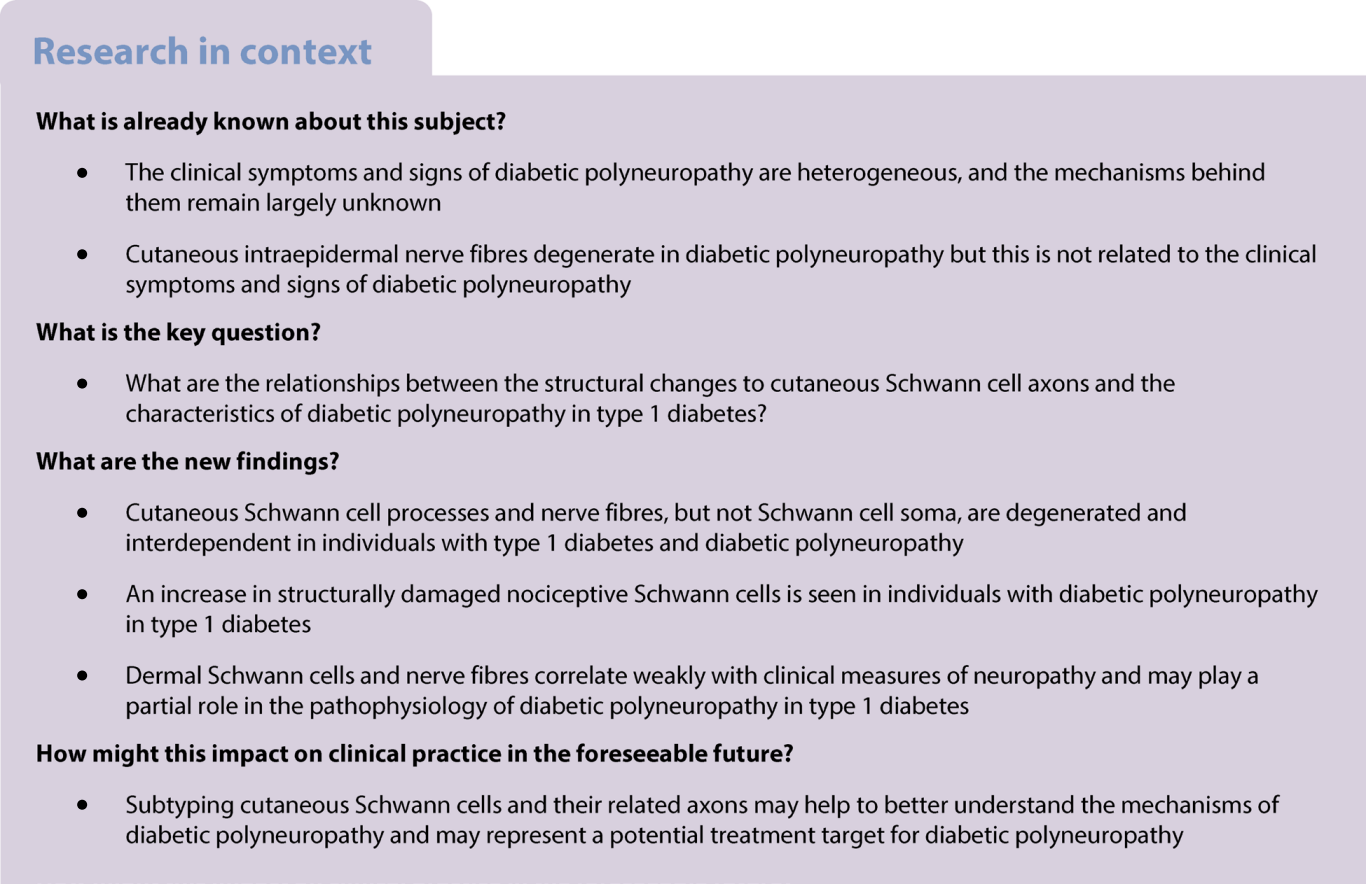


## Introduction

Diabetic polyneuropathy (DPN) is one of the most common complications of diabetes [1, 2], and is characterised by sensory dysfunction and in some cases neuropathic pain [3–5]. However, the mechanisms behind DPN and painful DPN are not fully understood.

Axonal degeneration and functional loss have been found both in myelinated and unmyelinated peripheral nerve fibres in DPN [6–8], and the decreased cutaneous free nerve endings measured by intraepidermal nerve fibre density (IENFD) is an important confirmation in DPN diagnosis [9–11]. However, previous studies have reported that the reduction of nociceptors and their morphological abnormalities correlate poorly with symptoms of DPN and are unlikely to be a major contributor to the development of painful neuropathy [12, 13]. There is, therefore, a need to identify other potential markers that may explain why some individuals with diabetes, but not all, develop DPN.

As one of the main glia cell types in the peripheral nervous system (PNS), Schwann cells (SCs) not only provide vital neurotrophic factors for peripheral nerves but also serve as the main resource for axonal myelination in PNS [14, 15]. The existence and structural–functional normality of SCs are important for maintaining the integrity of peripheral nerves, thus making the SC a potential important factor in axonal degeneration and possibly development of symptoms of DPN [15–18].

A recent preclinical study reported a specialised cutaneous SC type, a nociceptive SC (nSC), which contributes to pain initiation [19]. A follow-up study showed a depletion of either nociceptors or nSCs in mouse skin after hydroxytamoxifen painting, which resulted in hyperalgesia induced by thermal and mechanical stimulations, suggesting a structural–functional relationship between nSCs and neuropathic symptoms in these animals [20]. Furthermore, our previous study found an increase in nSC number density in the hind-paw glabrous skin in a mouse model of type 1 diabetes, with hypersensitive behaviours stimulated by thermal and mechanical tests [21].

Here, we investigated structural changes in SCs and their axons in DPN and their association with symptoms of neuropathy. We quantified the expression level of SCs and peripheral nerves in skin biopsies taken from healthy control individuals and from well-characterised individuals with type 1 diabetes, including those without DPN, those with painless DPN and those with painful DPN.

## Methods

### Study participants and clinical diagnosis of DPN and pain

The participants included in this study were part of a larger cross-sectional study of 216 participants with type 1 diabetes and healthy controls without diabetes recruited at three Steno Diabetes Centers in Denmark (in Aarhus, Odense and Copenhagen) from 2019–2021. We randomly chose 20 participants from each group (healthy control individuals, individuals with type 1 diabetes without DPN [T1D], individuals with type 1 diabetes with painless DPN [T1DPN] and individuals with type 1 diabetes with painful DPN [P-T1DPN]) and added five to eight additional individuals solely based on self-reported sex and age to each group to minimise difference between the groups. Inclusion criteria for all participants were a confirmed diagnosis of type 1 diabetes for at least 5 years and age between 30 and 75 years. Exclusion criteria for all participants were inability to understand oral instructions, neuropathy with causes other than diabetes, severe pain with causes other than neuropathy, any history of alcohol (>14 or >21 units/week for women and men, respectively) or substance abuse, pregnancy, any skin disorder or chronic ulcer in biopsy area, clinical intermittent claudication, non-palpable foot pulses and known allergy to lidocaine. Inclusion criterion for healthy control individuals was age between 30 and 75 years. Exclusion criteria for healthy control individuals were the same as for the other groups, except that no neurological or metabolic disorders were allowed, neither were clinical depression and ongoing pain of any cause. It is unknown how well the study sample represents the source population in terms of sex, ethnicity, age, regional and socioeconomic factors. However, given the inclusion criteria, we only assessed individuals aged 30–75 years and the individuals were recruited at Steno Centers across Denmark. No information on ethnicity or race was collected, as this information was not believed to have any influence on the study outcome. The study was open to individuals of all races, sex and socioeconomic status.

Anthropometric measures were collected and comprehensive evaluation was conducted on all study participants. Ankle and knee reflexes were tested, and vibration detection threshold on the hallux was measured using a biothesiometer (Horwell Neurothesiometer; Scientific Laboratory Supplies, Nottingham, UK). Values over 25 V were considered abnormal. Mechanical detection threshold was assessed using a 10 g monofilament (Neuropen; Owen Mumford, Woodstock, UK) under the hallux, and a decreased sensation was indicated if seven or fewer out of ten stimuli were detected. Pinprick sensation was tested using Neurotip (Owen Mumford) on the hallux, and temperature sensation was evaluated using thermo-rollers of 25°C (cold) and 40°C (warmth) on top of the hallux (Rolltemp II, Somedic, Sösdala, Sweden). Participants were asked to indicate whether the rollers felt cold, warm or neutral. Additionally, the participants were assessed using DPNCheck (NeuroMetrix, Woburn, MA, USA), which is a surrogate for a full nerve conduction study and measures the amplitude and velocity of the sural nerve. Amplitude < 4 µV and velocity < 40 m/s were considered abnormal. A punch skin biopsy of 3 mm was taken and a panel of blood biomarkers was analysed. The Toronto Diabetic Neuropathy Expert Group criteria were used to diagnose DPN, through evaluation of a combination of symptoms and signs [10]. Signs of DPN were assessed using the tests described above, and the diagnosis was confirmed through abnormal IENFD results from skin biopsy and DPNCheck. Participants who had a diagnosis of diabetes, reported pain in both feet/legs, had abnormal sensory signs in the feet/legs and abnormal IENFD or DPNCheck results were considered to have neuropathic pain. The severity of DPN was determined by using the Toronto clinical neuropathy score (TCNS) and the level of pain was assessed using a numeric rating scale (NRS; only participants scoring 0 [non-painful DPN] or >3 [painful DPN] on the 0–10 NRS were included in the study) and the Neuropathic Pain Symptom Inventory questionnaire (NPSI).

The study was performed in accordance with the Helsinki Declaration II and had the approval from the Regional Ethics Committee as well as the Danish Data Protection Agency (1-10-72-103-19). All participants gave their voluntary consent following a detailed explanation of the study procedures.

### Skin biopsy

Punch skin biopsies (Miltex, York, PA, USA) (3 mm), were taken at the distal leg (10 cm above the lateral malleolus) under sterile conditions following intradermal injection of 1% lidocaine, in accordance with published guidelines [22, 23]. Zamboni’s fixative and 20% (wt/vol.) sucrose (in 0.1 mol/l phosphate buffer) were used for the fixation (overnight) and cryoprotection (overnight) of the specimens. After snap freezing, all specimens were stored at −20°C until further processing. The experimenters were blinded to the origin of the skin biopsies during the immunofluorescent staining and quantification of skin biopsy measures.

### Immunofluorescent staining

Skin sections (50 µm thick) were cut and transferred to a neutral PBS buffer in 48-well plates. Target retrieval (in 10 mmol/l sodium citrate buffer for 20 min at 80°C) and blocking (in 1% [wt/vol.] BSA, 0.3% [vol./vol.] Triton X-100 in PBS solution for 1 h) were performed before immunolabelling.

For the primary antibodies, SCs were labelled by rabbit anti-S100 calcium-binding protein B (S100B; the antibody is ready to use with no dilution; DAKO, Denmark) and goat anti-transcription factor SOX10 antibodies (1:100 in S100B antibody solution [ready to use], R&D system, UK), and mouse anti-PGP9.5 antibody (1:1000 in S100B antibody solution [ready to use]; Bio-Rad, USA) was used to label peripheral nerves. Skin sections were incubated with the three primary antibodies in blocking buffer over two nights at 4°C, followed by a 1.5 h incubation in secondary antibody solution. The secondary antibodies were used at a concentration of 1:650 in blocking buffer: Alexa Fluor 488 donkey anti-mouse IgG (H+L) (Abcam, UK); Alexa Fluor 647 donkey anti-rabbit IgG (H+L) (Invitrogen, USA); and Alexa Fluor 594 donkey anti-goat IgG (H+L) (Invitrogen, Waltham, MA, USA) at room temperature. Lastly, DAPI (1:5000 in PBS buffer; Sigma, St Louis, MI, USA) nucleus counterstaining was applied to the skin sections before mounting.

### Imaging

Z-stack images (21 µm thick, with a 1.5 µm interval between images) were acquired with a ×40 lens using a laser-scanning spectral confocal microscope (LSM 800; Zeiss, Germany). The tile function from the ZEN microscopy software (Zeiss, Germany) was used to stitch single field images into one large image that included the entire region of interest (ROI). A minimum of two Z-stack images (15 stacks with a thickness of 21 µm) including the ROI in two individual skin sections from the same participant were captured and used for all quantitative analysis.

### Quantification

All quantifications were performed on orthogonally projected images of the original Z-stack confocal images. Dermal SC (DSC) area fraction and dermal nerve fibre (DNF) area fraction were determined from orthogonal average-projection images of the original Z-stack confocal images using FIJI (University of Wisconsin, WI, USA). Other quantifications were analysed on the orthogonal max-projection images of the original Z-stack confocal images using ZEN 3.3 (blue edition, Zeiss, Germany).

#### SC quantification

nSCs were identified as S100^+^/SOX10^+^/DAPI^+^ cells localised within 25 µm below the epidermal–dermal border but excluding glands and large nerve bundles as in our previous study [21]. The number density of nSCs abutting to nerve fibres (nSC+nerve complex; PGP9.5^+^/S100^+^/SOX10^+^/DAPI^+^) and the number density of nSCs not abutting to nerve fibres (nSC−nerve complex; PGP9.5^−^/S100^+^/SOX10^+^/DAPI^+^) were estimated (for details see electronic supplementary material [ESM] Methods).

The total DSC expression level was estimated by measuring the DSC area fraction, which was estimated by measuring the DSC-occupied area, divided by the total area of the ROI in the dermal area within 200 µm below the epidermal–dermal border from the orthogonally projected 2D confocal images (for details see ESM Methods).

#### Peripheral nerve fibre quantification

IENFD was counted following published guidelines [22, 24].

The DNF expression level was estimated by measuring the DNF area fraction, the same method as used for measuring DSC area fraction (for details see ESM Methods).

#### Comparison between methods for DNF and SC quantifications

To compare the results with previously published methods, we analysed the expression of subepidermal SCs (including the subepidermal SC process [SSCP]) and subepidermal SC soma [SSCS]) and subepidermal nerve fibres (SNFs) using a method optimised from a published study [25]. Here, we quantified the solitary and total SSCS number density, SSCP density and SNF density per volume within 40 µm from the border (for details see ESM Methods), and per length within 40 µm from the border (the original method [25]).

### Statistical analysis

Power calculation for sample size was based on expected moderate effect sizes (Cohen’s *d*=0.6), 80% power and α of 0.5, resulting in approximately 20–25 individuals needed in each group to show group differences in most skin biopsy measurements. Continuous data are presented as means ± SD or medians (IQR) according to distribution. Categorical data are presented as frequency with percentage. Non-normally distributed continuous data were normalised using square root transformation, when appropriate (determined by QQ plots). Comparisons between multiple groups were performed using either one-way ANOVA (parametric test) or Kruskal–Wallis equality-of-populations rank test (non-parametric test). ANCOVA analysis was performed adjusting for sex, age and HbA_1c_, providing adjusted *p* values. Fisher’s exact test was applied when comparing categorical variables. Co-linearity was checked by observing the variance inflation factor (VIF), which was deemed to show high correlation between two explanatory variables if >5 (this was not the case). Post hoc multiple comparisons were performed on variables showing significant differences. Parametric data were analysed with pairwise comparisons *t* tests adjusting for age, sex and HbA_1c_, and results were presented as reverse-transformed means with 95% CI and *p* values. Dunn’s test was used for post hoc multiple comparisons tests of non-parametric data, and the results were presented as *p* values (not adjusted for confounders). Both of the post hoc tests were corrected for multiple testing using Bonferroni correction. Linear correlation was carried out using Pearson’s correlation analysis. Finally, partial correlational analysis was performed by adjusting for age and sex, and HbA_1c_. Statistical significance (α) was set at a *p* value ≤0.05.

## Results

### Participant characterisation

A total of 107 participants consisting of 25 individuals with type 1 diabetes without DPN, 30 with type 1 diabetes and painless DPN, 27 with type 1 diabetes and painful DPN and 25 healthy control individuals, were included. Main clinical characteristics are described in Table 1. There were statistically significant differences between the groups in age, total cholesterol, diastolic BP, eGFR, HbA_1c_, LDL-cholesterol and urinary albumin/creatinine ratio. In the confirmatory tests of DPN, differences were seen in IENFD from skin biopsies and nerve conduction velocity and amplitude using DPNCheck between the groups (all *p*<0.001), and sensory tests (mechanical, vibration and thermal sensation) also differed between the groups (all *p*<0.001). Most of the participants with DPN showed both small- and large-fibre abnormalities, indicating presence of mixed fibre neuropathy. Median (IQR) pain intensity (assessed by NRS) was 5.0 (5.0–6.0) and 5.0 (5.0–7.0) in P-T1DPN at 24 h and 7 days, respectively, before the sensory test and skin biopsy acquisition.

**Table 1:** Participant characteristics

Characteristic	Control (*n*=25)	T1D (*n*=25)	T1DPN (*n*=30)	P-T1DPN (*n*=27)	*p* value
Female sex	12 (48.0)	14 (56.0)	13 (43.3)	15 (55.6)	0.760
Diabetes duration, years	–	30.3±14.6	40.3±15.7	32.9±10.1	0.023*
Age, years	59.3 (50.6–64.5)	54.4 (46.2–61.0)	63.4 (57.9–71.1)	58.8 (52.6–63.3)	0.018*
BMI, kg/m^2^	27.8 (24.5–31.8)	26.3 (24.3–28.3)	27.0 (23.3–30.8)	27.9 (23.8–31.2)	0.775
Smoking today	0 (0.0)	0 (0.0)	1 (3.3)	3 (11.1)	0.163
Alcohol, ≥8 units/week	4 (18.2)^a^	5 (20.0)	10 (33.3)	4 (14.8)	0.392
Exercise, >1 times/week	20 (90.9)^a^	21 (84.0)	20 (66.7)	20 (74.1)	0.175
Antihypertensive medication	1 (4.0)	12 (48.0)	25 (83.3)	16 (59.3)	<0.001***
Cholesterol-lowering medication	1 (4.0)	16 (64.0)	28 (93.3)	22 (81.5)	<0.001***
IENFD, nerves/mm	4.6 (3.2–5.3)	2.7 (1.6–3.9)	0.5 (0.0–1.7)	0.5 (0.0–2.5)	<0.001***
DPNCheck, abnormal	4 (16.7)^a^	4 (16.7)^a^	29 (96.7)	22 (81.5)	<0.001***
DPNCheck, amplitude, μV	8.5 (6.0–11.5)	9.0 (5.5–12.5)	2.0 (0.0–4.0)	3.0 (2.0–5.0)	<0.001***
DPNCheck, conduction velocity, m/s	53.5 (49.5–58.0)	47.5 (43.5–51.0)	34.0 (0.0–44.0)	37.5 (25.0–43.0)	<0.001***
Neuropen, abnormal	0 (0.0)	0 (0.0)	19 (63.3)	12 (44.4)	<0.001***
Neurotip, abnormal	1 (4.0)	1 (4.0)	14 (46.7)	12 (44.4)	<0.001***
Warmth, abnormal	0 (0.0)	1 (4.0)	19 (63.3)	14 (51.9)	<0.001***
Cold sensation, abnormal	1 (4.0)	1 (4.0)	21 (70.0)	20 (74.1)	<0.001***
Biothesiometer >25 V	0 (0.0)	2 (8.0)	18 (60.0)	16 (59.3)	<0.001***
Biothesiometry (V)	6.7 (5.6–9.8)	10.2 (5.8–15.0)	27.6 (18.3–43.0)	30.8 (17.3–50.0)	<0.001***
Pain intensity in the last 24 h, NRS score	−	0.0 (0.0–0.0)	0.0 (0.0–0.0)	5.0 (5.0–6.0)	0.019*
Pain intensity in the last 7 days, NRS score	−	0.0 (0.0–0.0)	0.0 (0.0–0.0)	5.0 (5.0–7.0)	0.009**
MNSI-Q score	2.0 (2.0–3.0)	3.0 (2.0–4.0)	5.0 (4.0–6.0)	7.0 (6.0–9.0)	<0.001***
MNSI clinical score	0.0 (0.0–1.0)	1.0 (0.0–1.0)	4.5 (3.0–6.0)	4.5 (3.0–6.0)	<0.001***
TCNS score	0.0 (0.0–1.0)	1.0 (0.0–2.0)	6.0 (4.0–9.0)	9.0 (7.0–11.0)	<0.001***
Systolic BP, mmHg	141.7 (132.0–147.7)	138.7 (129.7–149.3)	134.3 (128.7–152.0)	140.0 (130.7–150.3)	0.963
Diastolic BP, mmHg	86.0 (78.3–91.3)	76.7 (72.7–83.0)	71.3 (66.7–81.3)	75.7 (72.3–84.3)	<0.001***
HbA_1c_, mmol/mol	35.8 (34.6–38.7)	55.0 (49.0–58.5)	62.6 (57.0–68.1)	65.0 (55.6–71.0)	<0.001***
HbA_1c_, %	5.4 (5.3–5.7)	7.2 (6.6–7.5)	7.9 (7.4–8.4)	8.1 (7.2–8.6)	<0.000***
Glucose, mmol/l	5.5 (5.1–6.2)	8.6 (6.8–10.1)	8.3 (6.3–11.6)	9.3 (5.7–14.3)	<0.001***
Triacylglycerol, mmol/l	1.2 (0.9–1.7)	0.8 (0.7–1.3)	1.0 (0.6–1.6)	1.0 (0.7–1.2)	0.104
Total cholesterol, mmol/l	5.6 (5.2–5.7)	4.3 (3.9–5.0)	4.2 (3.3–4.7)	4.3 (3.7–4.6)	<0.001***
LDL-cholesterol, mmol/l	3.2±0.7	2.2±0.7	1.9±0.7	2.0±0.6	<0.001***
HDL-cholesterol, mmol/l	1.6 (1.3–1.9)	1.8 (1.5–2.1)	1.7 (1.3–1.9)	1.6 (1.4–1.9)	0.401
eGFR, ml/min per 1.73 m^2^	90.0 (84.5–90.0)	90.0 (85.7–90.0)	84.1 (62.0–90.0)	90.0 (86.0–98.0)	0.025*
Urine albumin/creatinine ratio, mg/mmol	1.1 (0.5–1.7)	0.7 (0.4–1.2)	2.4 (0.9–4.7)	1.4 (0.7–3.3)	0.010*

Categorical variables are presented as *n* (%); continuous data are presented as median (IQR) for non-parametric variables and as mean ±SD for parametric variables

Fisher’s exact test was carried out for categorical variables. For continuous variables, one-way ANOVA (parametric variables [diabetes duration, LDL-cholesterol]) or Kruskal–Wallis H test (non-parametric variables) were used

^a^Data were missing for a few participants as follows: control group – Alcohol, ≥8 units/week, *n*=3; Exercise, >1 times/week, *n*=3; DPNCheck, abnormal, *n*=1; T1D group – DPNCheck, abnormal, *n*=1

*p<0.05, **p<0.01 and ***p<0.001

MNSI, Michigan Neuropathy Screening Instrument; MNSI-Q, MNSI questionnaire

### Visualisation and quantification of SCs

**Fig. 1:**
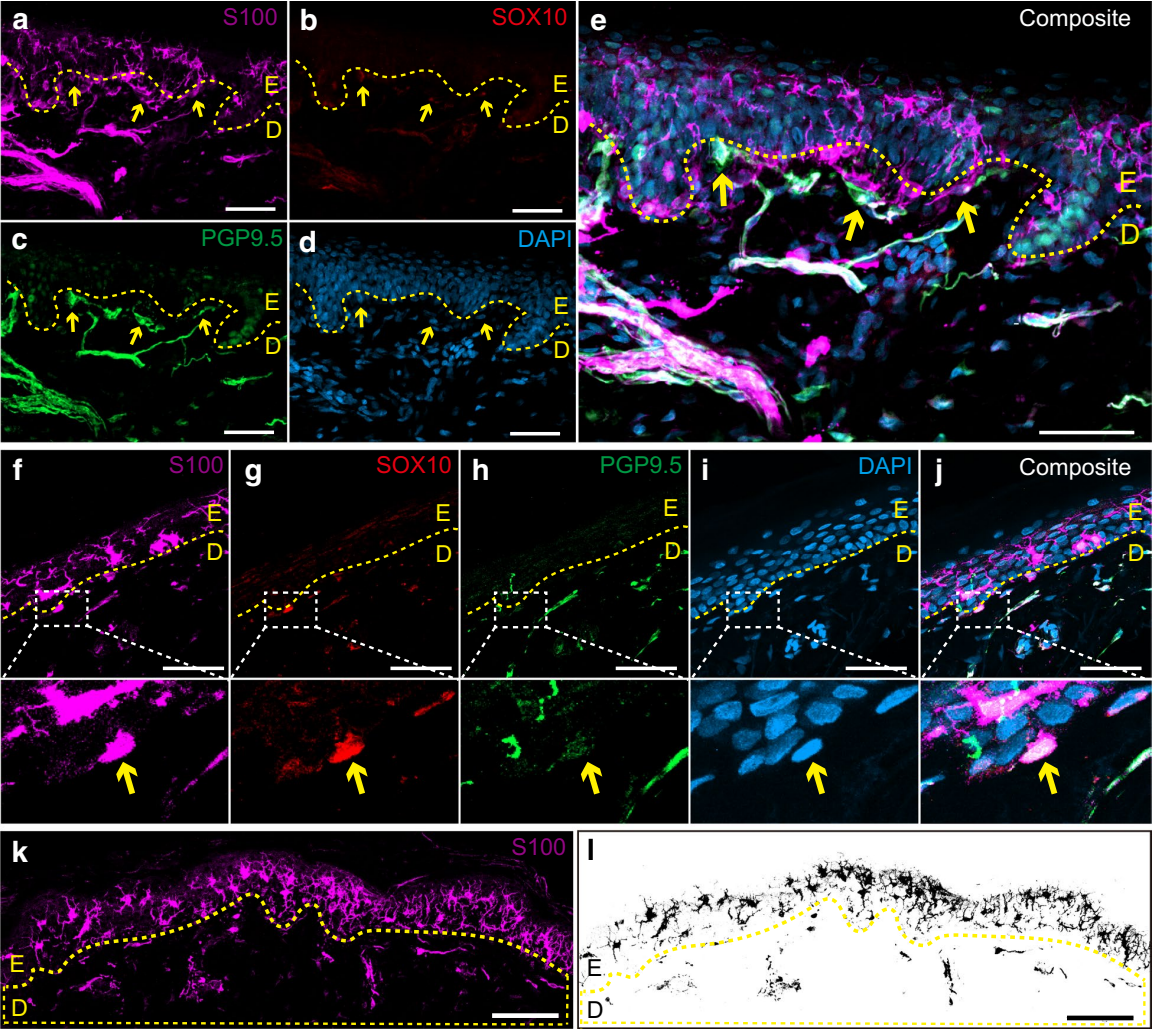
Representative immunofluorescent visualisation of peripheral nerves and nSCs in human skin. (**a**–**e**) A representative structure of nSC+nerve complex: (**a**) S100 antibody-labelled overall DSCs and epidermal Langerhans cells (magenta); (**b**) SOX10-positive nuclei (red); (**c**) PGP9.5 antibody-labelled peripheral nerves (green); (**d**) nuclei labelled by DAPI (blue); (**e**) composite image. (**f**–**j**) A representative structure of nSC–nerve complex: (**f**) S100 antibody-labelled overall DSCs and epidermal Langerhans cells (magenta); (**g**) SOX10-positive nuclei (red); (**h**) PGP9.5 antibody-labelled peripheral nerves (green); (**i**) nuclei labelled by DAPI (blue); (**j**) composite image. (**k**) S100^+^ SCs in dermal ROI. (**l**) Filtered positive signals of DSCs. Scale bars, 50 μm (**a**–**j**) and 100 μm (**k**, **l**). Arrows indicate the expression of different markers of nSC or nerve fibre in the subepidermal area; yellow dotted lines indicate the epidermal–dermal border. D, dermal; E, epidermal

**Fig. 2:**
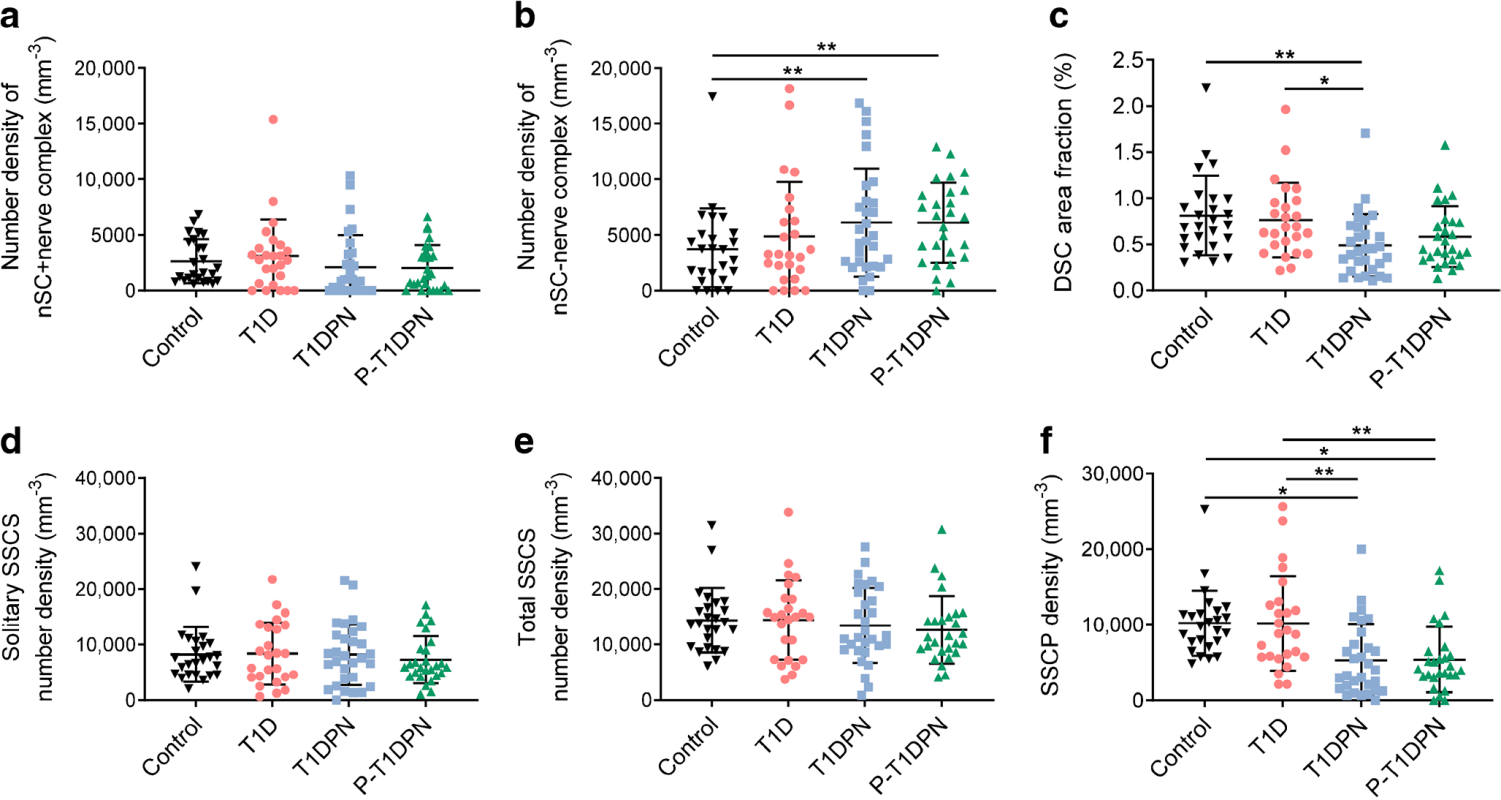
Quantification of DSCs in the skin. (**a**) Number density of nSCs abutting to nerve fibres (nSC+nerve complex). (**b**) Number density of nSCs not abutting to nerve fibres (nSC−nerve complex). (**c**) DSC area fraction in each group. (**d**) Solitary SSCS number density. (**e**) Total SSCS number density. (**f**) SSCP density. Post hoc analysis: parametric (square-root transformed) data are presented as adjusted *p* value and mean difference with 95% CI analysed by pairwise comparisons *t* tests, adjusting for age, sex and HbA_1c_ (**b**, **f**); non-parametric data are presented as non-confounder adjusted *p* value by using Dunn’s test (**c**); *p<0.05 and **p<0.01

Nociceptors and nSCs were identified in the subepidermal area in human hairy skin (Fig. 1a–e and ESM Fig. 1) but they do not always form a glia–neuro complex as in mouse glabrous skin [19, 21]; for examples, see Fig. 1f–j. Conversely, most nSCs did not abut to nerve fibres (nSC–nerve complex), with the median percentage of nSCs abutting to nerve fibres (nSC+nerve complex) being <50% in all groups (Table 2). We found no difference in the number density of nSC+nerve complexes between the four groups (*p*<0.148, *p*_adj_=0.551; Fig. 2a, Table 2). However, there was a difference in the number density of nSC–nerve complexes between the groups after adjusting for age, sex and HbA_1c_ (*p*=0.078, *p*_adj_=0.004; Fig. 2b, Table 2). Additionally, post hoc analysis revealed a significant increase of nSC–nerve complexes in the T1DPN and P-T1DPN groups compared with the healthy control group after adjusting for age, sex and HbA_1c_ (both *p*_adj_<0.01), but no significant difference was seen between the other two groups (all *p*_adj_>0.05; Table 3).

**Table 2:** Cutaneous SC and nerve fibre quantifications and comparisons in the four study groups

Quantification method	Control (*n*=25)	T1D (*n*=25)	T1DPN (*n*=30)	P-T1DPN (*n*=27)	*p* value	*p*_adj_ value
Percentage of nSCs+nerve, %	39.6 (28.1–50.7)	32.7 (27.1–66.7)	12.8 (0.0–37.6)	23.5 (0.0–43.7)	0.002**	−
Number density of nSCs+nerve complex, mm^−3^	1598.9 (902.7–4351.3)	2773.5 (648.1–3806.6)	940.6 (0.0–3273.8)	1319.5 (0.0–3512.4)	0.148	0.551
Number density of nSC−nerve complex, mm^−3^	3684.0 (1552.2–5051.7)	3268.8 (1916.0–6234.8)	4701.5 (2214.7–8034.1)	6602.2 (3009.2–8855.5)	0.078	0.004**
DSC area fraction, %	0.7 (0.6–1.0)	0.7 (0.5–1.0)	0.4 (0.2–0.7)	0.4 (0.3–0.7)	0.003**	−
Solitary SSCS number density, mm^−3^	7212.7 (4580.3–10,224.3)	8155.2 (4195.6–13,109.9)	7792.4 (3870.4–11,723.5)	5961.5 (4415.6–9214.4)	0.907	0.911
Total SSCS number density, mm^−3^	13,782.9 (9590.0–16,658.2)	15,000.5 (7309.7–18,192.4)	11,421.0 (9083.2–19,377.6)	11,379.9 (8849.9–15,021.0)	0.683	0.962
SSCP density, mm^−3^	9822.2 (7745.2–11,457.4)	8846.8 (5759.8–12,607.9)	3642.2 (1554.3–7377.3)	4144.9 (3151.2–6498.3)	<0.001***	<0.001***
DNF area fraction, %	0.9 (0.8–1.2)	0.8 (0.6–1.1)	0.6 (0.5–0.9)	0.6 (0.4–0.8)	0.001**	−
SNF density, mm^−3^	11,077.9 (8073.7–12,644.4)	10,823.9 (6355.5–12,833.6)	3926.2 (1855.2–6916.3)	4973.7 (3231.4–8162.8)	<0.001***	<0.001***

All continuous variables were non-normally distributed and are presented as median (IQR)

After square root transformation, variables were analysed using both ANOVA (*p* value) and ANCOVA (*p*_adj_ value, adjusting for age, sex and HbA_1c_). Variables that could not be transformed were analysed by Kruskal–Wallis H test (non-adjusted *p* value)

*p<0.05, **p<0.01 and ***p<0.001

**Table 3:** Post hoc comparisons of skin biopsy quantifications between four study groups

Quantification	Mean difference	95% CI (lower)	95% CI (upper)	*p* value	*p*_**adj**_ value
Number density of nSC−nerve complexes, mm^**−**3^					
T1D vs Control	465.3	−64.1	2616.2	–	0.315
T1DPN vs Control	1774.5	66.1	5794.9	–	0.007**
P-T1DPN vs Control	1863.4	91.1	5897.0	–	0.005**
T1DPN vs T1D	422.5	−31.1	2179.3	–	0.22
P-T1DPN vs T1D	466.4	−14.1	2204.7	–	0.144
P-T1DPN vs T1DPN	1.1	−501.0	598.6	–	1.000
DSC area fraction, %					
T1D vs Control	–	–	–	1.000	–
T1DPN vs Control	–	–	–	0.003**	–
P-T1DPN vs Control	–	–	–	0.105	–
T1DPN vs T1D	–	–	–	0.011*	–
P-T1DPN vs T1D	–	–	–	0.242	–
P-T1DPN vs T1DPN	–	–	–	0.765	–
SSCP density, mm^−3^					
T1D vs Control	−26.1	−1025.4	475.4	–	1.000
T1DPN vs Control	−1209.3	−4317.8	−14.7	–	0.019*
P-T1DPN vs Control	−1230.7	−4313.2	−20.1	–	0.016*
T1DPN vs T1D	−880.0	−2855.7	−34.7	–	0.007**
P-T1DPN vs T1D	−898.3	−2813.6	−47.6	–	0.004**
P-T1DPN vs T1DPN	−0.1	−467.4	441.2	–	1.000
DNF area fraction, %					
T1D vs Control	–	–	–	1.000	–
T1DPN vs Control	–	–	–	0.009**	–
P-T1DPN vs Control	–	–	–	0.002**	–
T1DPN vs T1D	–	–	–	0.136	–
P-T1DPN vs T1D	–	–	–	0.048*	–
P-T1DPN vs T1DPN	–	–	–	1.000	
SNF density, mm^−3^					
T1D vs Control	−44.5	−1185.7	445.0	–	1.000
T1DPN vs Control	−1579.3	−5134.5	−61.2	–	0.007**
P-T1DPN vs Control	−1319.8	−4609.4	−22.7	–	0.015*
T1DPN vs T1D	−1093.7	−3317.5	−73.0	–	0.003**
P-T1DPN vs T1D	−879.7	−2858.3	−34.3	–	0.007**
P-T1DPN vs T1DPN	11.6	−345.1	645.1	–	1.000

Parametric (square-root transformed) data are presented as adjusted *p* value and mean difference with 95% CI analysed by pairwise comparisons *t* tests adjusting for age, sex and HbA_1c_ and non-parametric data are presented as non-confounder adjusted *p* value by using Dunn’s test

*p<0.05, **p<0.01

We then visualised and quantified the overall expression of DSCs localised within 200 µm below the dermal–epidermal border (Fig. 1k,l). There was a difference in DSC area fraction between the groups (*p*=0.003; Fig. 2c, Table 2). We found a statistically significant decline in DSC area fraction in the T1DPN group compared with the healthy control and T1D groups (both *p*<0.05; Table 3). Declines, albeit not statistically significant, were seen in the P-T1DPN group compared with the healthy control and T1D groups (Table 3).

#### Comparison between methods

To allow for a direct method comparison, we also quantified the SSCS and SSCP density using a quantification method that was modified from a recently published study [25]. There was no difference in either solitary or total SSCS number density between the groups, both before and after adjusting for age, sex and HbA_1c_ (all *p and p*_adj_>0.05; Fig. 2d,e; Table 2). However, a significant difference in SSCP density was seen between the four groups (*p*<0.001, *p*_adj_<0.001; Fig. 2f; Table 2), and post hoc analysis showed declines of SSCP density in the T1DPN and P-T1DPN groups compared with the healthy control and T1D groups after adjusting for confounders (all *p*_adj_<0.01; Table 3). We also quantified solitary and total SSCS number density and SSCP density per length using the methods published in the aforementioned study [25], and the results pointed to the same conclusion (data not shown).

### Visualisation and quantification of cutaneous nerve fibres

**Fig. 3:**
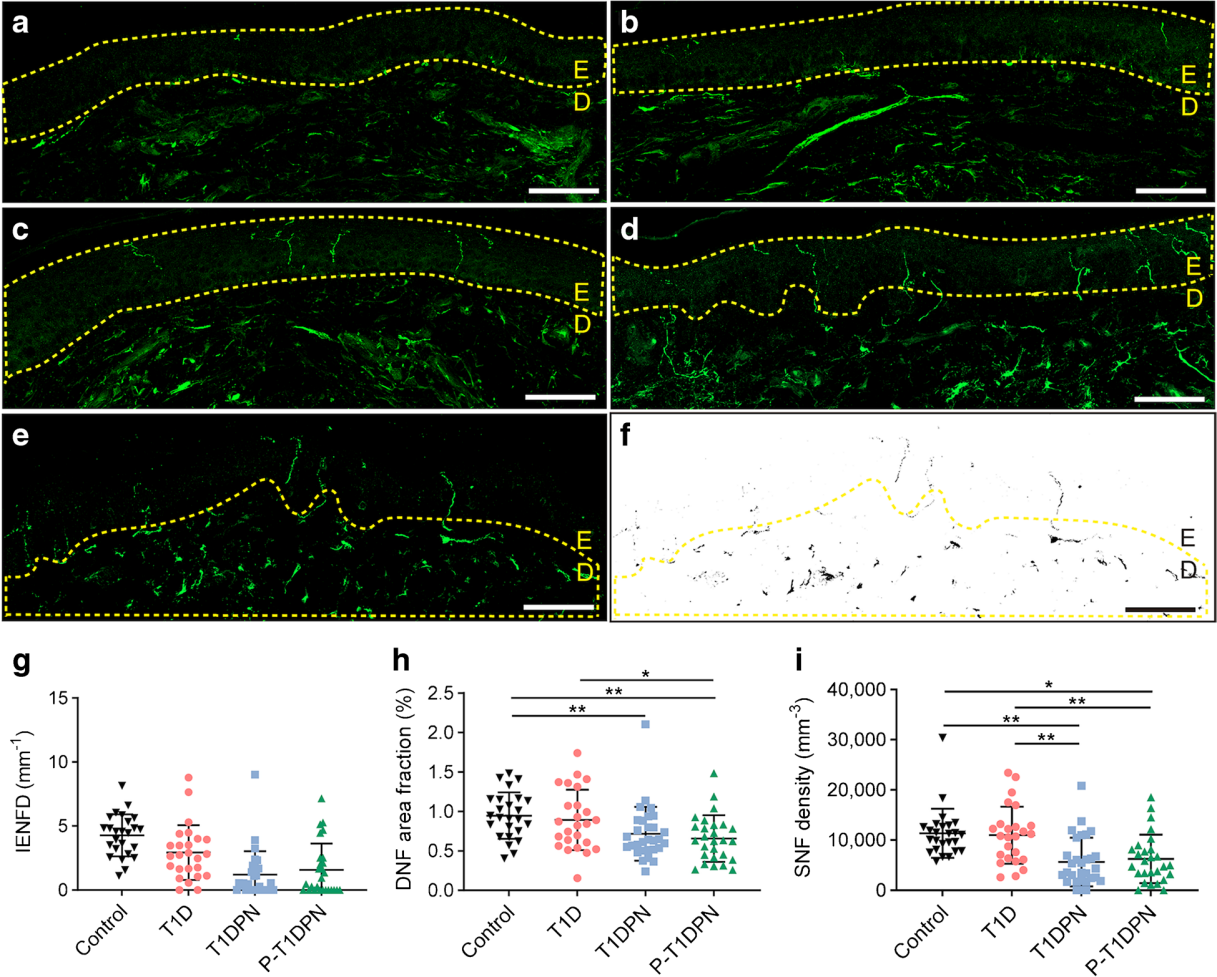
(**a**–**d**) Representative images of cutaneous nerve fibres (PGP9.5^+^) in participants in the P-T1DPN (**a**), T1DPN (**b**), T1D (**c**) and healthy control (**d**) groups. (**e**, **f**) Representative images for DNF occupancy analysis: (**e**) the ROI for DNF occupancy analysis; (**f**) the filtered positive signals of peripheral nerves within the ROI. (**g**) IENFD in each group (post hoc analysis was not performed for IENFD because IENFD was used in participant classification). (**h**) DNF area fraction in each group. (**i**) SNF density in each group. Scale bar, 100 μm (**a**–**f**). The yellow dotted line in (**a**–**d**) marks the border of the epidermis; in (**e**, **f**) the line marks the ROI for analysing the DNF occupancy. Post hoc analysis: parametric (square-root transformed) data are presented as adjusted *p* value and mean difference with 95% CI analysed by pairwise comparisons *t* tests, adjusting for age, sex and HbA_1c_ (**h**); non-parametric data are presented as non-confounder adjusted *p* value by using Dunn’s test (**i**); *p<0.05 and **p<0.01. D, dermal; E, epidermal

Representative images of free nerve endings for each group are shown in Fig. 3a–d; quantification of IENFD is shown in Fig. 1g and Table 1. Post hoc analysis was not performed for IENFD as it was used in participant classification. The overall expression of DNFs was assessed by measuring DNF area fraction in the dermal area within 200 µm below the dermal–epidermal border, as illustrated in Fig. 3e,f. A difference in DNF area fraction was seen between the groups (*p*=0.001; Fig. 3h; Table 2). Post hoc group comparison showed no significant difference in DNF area fraction between participants with painless and painful DPN (*p*=1.000), but there was a decrease in the P-T1DPN group compared with the healthy control and T1D groups (both *p*<0.05), and a decrease in the T1DPN group compared with the control group (*p*=0.009; Table 3).

#### Comparison between methods

A significant difference in SNF density per volume (a method modified from a published study [25]) was seen between the groups (*p*<0.001, *p*_adj_<0.001; Fig. 3i; Table 2). Post hoc analysis showed reduced SNF density per volume in the T1DPN and P-T1DPN groups compared with the healthy control and T1D groups (all *p*_adj_<0.05), while there was no difference between the T1DPN and P-T1DPN groups (*p*_adj_=1.000, Table 3). Lastly, quantifying SNF density per length using the previously published method was also performed [25] and the results pointed towards the same conclusion (data not shown).

### Association between cutaneous SCs and nerve fibres

To investigate the association between cutaneous SCs and nerve fibres, we performed correlational analysis between the skin biopsy quantifications (Table 4).

**Table 4:** Pearson’s *r* correlations between the quantifications of cutaneous SCs and nerve fibres in all participants

Correlation analysis	DNF area fraction (%)	DSC area fraction (%)	Number density of nSCs+nerve (mm^−3^)	Number density of nSC−nerve (mm^−3^)	SNF density (mm^−3^)	SSCP density (mm^−3^)	Solitary SSCS number density (mm^−3^)	Total SSCS number density (mm^−3^)
DSC area fraction (%)								
*r*	0.58							
*p* value	<0.001***	–	–	–	–	–	–	–
Number density of nSC+nerve (mm^−3^)								
*r*	0.25	0.16						
*p* value	0.008**	0.107	–	–	–	–	–	–
Number density of nSC−nerve (mm^−3^)								
*r*	−0.02	0.015	0.19					
*p* value	0.838	0.875	0.048*	–	–	–	–	–
SNF density (mm^−3^)								
*r*	0.58	0.64	0.26	0.21				
*p* value	<0.001***	<0.001***	0.008**	0.033*	–	–	–	–
SSCP density (mm^−3^)								
*r*	0.63	0.65	0.26	−0.15	0.95			
*p* value	<0.001***	<0.001***	0.006**	0.122	<0.001***	–	–	–
Solitary SSCS number density (mm^−3^)								
*r*	0.37	0.17	0.47	0.57	0.13	0.24		
*p* value	<0.001***	0.076	<0.001***	<0.001***	0.197	0.015*	–	–
Total SSCS number density (mm^−3^)								
*r*	0.43	0.38	0.46	0.41	0.30	0.41	0.84	
*p* value	<0.001***	<0.001***	<0.001***	<0.001***	0.002**	<0.001***	<0.001***	–
IENFD (nerves/mm)								
*r*	0.38	0.44	0.36	−0.22	0.64	0.61	0.08	0.21
*p* value	<0.001***	<0.001***	<0.001***	0.020*	<0.001***	<0.001***	0.437	0.032*

Correlation results (no confounding adjustment) are presented as Pearson *r* and significance level (*p* values)

*p<0.05, **p<0.01 and ***p<0.001

SC measures, including the number density of nSC+nerve complexes, SSCP density, solitary and total SSCS density, correlated positively with each other, but the number density of nSC−nerve complexes did not correlate with DSC area fraction or SSCP density.

IENFD correlated positively with DNF area fraction, SNF density, DSC area fraction, number density of nSC+nerve complexes, SSCP density and total (but not solitary) SSCS number density, and correlated negatively with the number density of nSC−nerve complexes.

### Association between skin biopsy results and markers of neuropathy

Correlation analysis is shown in Table 5. The number density of nSC+nerve complexes had a weak negative correlation with the Michigan Neuropathy Screening Instrument (MNSI) total score and biothesiometer but no correlation was found after adjusting for confounders. The number density of nSC−nerve complexes had a weak and positive correlation with TCNS score after, but not before, adjusting for confounders. Total SSCS density had a weak negative correlation with MNSI total score and TCNS score but no correlation was observed after adjusting for confounders.

**Table 5:** Correlation analysis between skin biopsy quantifications and characteristics/clinical signs in all participants

Quantification	MNSI-Q		MNSI total score		TCNS		Biothesiometer	
	Correlation	Partial correlation	Correlation	Partial correlation	Correlation	Partial correlation	Correlation	Partial correlation
Number density of nSC+nerve (mm^−3^)								
*r*	−0.14	−0.08	−0.22	−0.12	−0.17	−0.02	−0.19	−0.09
*p* value	0.165	0.445	0.025*	0.240	0.087	0.832	0.049*	0.388
Number density of nSC−nerve (mm^−3^)								
*r*	0.15	0.15	0.12	0.15	0.18	0.25	0.16	0.19
*p* value	0.136	0.136	0.207	0.122	0.061	0.011*	0.104	0.051
DSC area fraction (%)								
*r*	−0.25	−0.16	−0.41	−0.34	−0.38	−0.29	−0.32	−0.22
*p* value	0.009**	0.103	<0.001***	<0.001***	<0.001***	0.003**	0.001**	0.025*
Solitary SSCS number density (mm^−3^)								
*r*	−0.10	−0.03	−0.14	−0.06	−0.11	−0.02	−0.07	0.01
*p* value	0.335	0.733	0.163	0.556	0.264	0.806	0.428	0.889
Total SSCS number density (mm^−3^)								
*r*	−0.16	−0.12	−0.22	−0.17	−0.20	−0.14	−0.15	−0.07
*p* value	0.098	0.230	0.025*	0.094	0.044*	0.144	0.134	0.468
SSCP density (mm^−3^)								
*r*	−0.40	−0.32	−0.48	−0.38	−0.52	−0.43	−0.39	−0.24
*p* value	<0.001***	0.001**	<0.001***	<0.001***	<0.001***	<0.001***	<0.001***	0.013*
DNF area fraction (%)								
*r*	−0.33	−0.25	−0.33	−0.23	−0.37	−0.27	−0.19	−0.04
*p* value	<0.001***	0.012*	0.001**	0.018*	<0.001***	0.007**	0.053	0.686
SNF density (mm^−3^)								
*r*	−0.41	−0.33	−0.49	−0.38	−0.52	−0.43	−0.41	−0.27
*p* value	<0.001***	0.001**	<0.001***	<0.001***	<0.001***	<0.001***	<0.001***	0.006**

Normal correlation and partial correlation results are presented as Pearson *r* and significance level (*p* values); the adjusted confounders included age, sex and HbA_1c_

*p<0.05, **p<0.01 and ***p<0.001

Moreover, DSC area fraction, SSCP density and SNF density were weakly and negatively correlated with the MNSI questionnaire (MSNI-Q), MNSI total score, TCNS and vibration test (biothesiometer), although the correlation between DSC area fraction and MNSI-Q lost significance after adjusting for confounders. Finally, DNF area fraction correlated negatively with MNSI-Q, MNSI total score and TCNS score but not with vibration test.

## Discussion

This study investigated structural changes in specialised nSCs, as well as the overall cutaneous SCs and axons in individuals with type 1 diabetes and assessed their association with DPN and its symptoms. Together with the three group comparison results (ESM Results and Tables 1, 2), we found the following: (1) there was no difference in the number density of nSC+nerve complexes between the groups but there was an increase in the number density of nSC−nerve complexes in individuals with DPN (regardless of pain status) vs healthy control individuals and those without DPN; (2) there was a degeneration of overall DSCs (DSC area fraction) and SSCP density (but not solitary or total SSCS number density), and a degeneration of both epidermal and dermal cutaneous nerve fibres (IENFD, DNF area fraction and SNF density), in individuals with DPN compared with healthy control individuals and those without DPN; (3) there was no difference in any of the skin biopsy measurements between individuals with DPN with and without pain, nor between individuals without DPN and healthy control individuals, indicating that the markers do not play a significant role in diabetes or painful DPN, but possibly do in DPN; and (4) the expression of overall SCs, SC processes and cutaneous nerves correlated weakly and negatively with clinical examinations, including MNSI-Q, MNSI total score, TCNS score and biothesiometer. Taken together, the findings suggest that the expression of cutaneous SCs (particularly processes) and nerves are both reduced and interdependent in this cohort of individuals with DPN and correlate weakly with neuropathy severity but not neuropathic pain.

These findings are of interest for several reasons. First, just as in previous mouse studies, we found that nociceptors and nSC processes are interdependent in diabetes, meaning that if one cell type degenerates or is damaged, the other one will degenerate as well [20, 21]. Second, individuals with DPN display an increase of nSC−nerve complexes compared with healthy control individuals, while at the same time they have degenerated cutaneous nerve fibres and SSCPs. It is unclear whether these specialised nSCs exhibit greater plasticity than other types following damage. Third, we showed that there were no significant differences in any of the skin biopsy measurements between individuals with DPN with and without pain but the degenerated cutaneous SCs and nerves were associated with DPN. Importantly, the overall SCs, SSCPs and cutaneous nerves correlated negatively, albeit weakly, with MNSI-Q, MNSI total score, TCNS and biothesiometer. These results indicate that, like cutaneous nerves, SC processes, but not SC somata and specialised nSCs, are to some degree associated with neuropathy measures in this cohort of individuals with type 1 diabetes. Fourth, the findings highlight that the common classification of SCs into myelin and non-myelin SCs (also known as Remak SCs) may be oversimplified and that it is important to differentiate between different subtypes of SCs and their function.

There is currently no consensus on how to define and quantify nSCs and DSCs, making comparisons between studies difficult. We, therefore, supplemented our method of quantification with a replication of the methods used in the other study quantifying subepidermal SC and axons [25]. Our methods for quantifying overall DSCs and nerves yielded slightly different results compared with the optimised method described in the previous study for quantifying subepidermal SCs and nerves. More profound degeneration was found in SC processes and in nerves in the subepidermal area (40 μm below the epidermal–dermal border) compared with the SCs and nerves in the overall dermal area (200 μm below epidermal–dermal border). However, quantification of subepidermal SCs and nerves using both the original and modified methods described in the published study and this present study pointed to the same conclusions, suggesting that both methods are reliable.

In our preclinical study in mice, nSCs were defined as being S100^+^ and SOX10^+^ and co-localised with peripheral nerve fibres to form a mesh-like network at the subepidermal area (within 25 µm below the epidermal–dermal border), with glia–neuro projections entering the epidermis [21]. Here we observed similar structures in the human skin but most of the nSCs were not co-localised with peripheral nerve fibres. The structural differences in nSCs when comparing mice and humans might be due to the difference in skin biopsy locations (glabrous skin biopsies in mice and hairy skin biopsies in humans). The lack of specific biomarkers for the specialised nSCs is a considerable challenge for understanding those glial–neuronal complexes. Additionally, caution should be used when translating the nSC quantification criteria from preclinical studies in glabrous skin to clinical studies in hairy skin.

The discussion of SC classification and nSC definition prompts a reconsideration of how heterogeneous cutaneous SCs interact with cutaneous nerves and respond to nerve damage, as well as their involvement in neuropathic pain. This knowledge will aid in identifying more specific treatment targets for peripheral nerve diseases [26]. A further question arising from this study is whether the reprogramming capacity of cutaneous SCs remains the same, and how the associated biomarkers (e.g. SOX10) are involved in nSCs following nerve damage caused by hyperglycaemia.

The study has several limitations. The participants with DPN included in this study had relatively mild symptoms; most had median pain intensity with only few having severe pain. This may have resulted in less pronounced differences between the groups in some of the measures, including the nSCs, and thereby possibly the study was underpowered. Additionally, we were unable to completely match the groups of participants according to sex and age. However, the influence of these confounding factors on the results was minimised through adjusting for these confounders. Lastly, the cross-sectional design of the study did not allow us to investigate whether it is the nociceptors or the SCs that are damaged first in type 1 diabetes. Despite these limitations, the study has strengths, including careful phenotyping of individuals according to recommended guidelines as well as a detailed assessment of nerve fibres and SCs and their morphology using different methods.

### Conclusion

Cutaneous SCs and nerve fibres are partially interdependent in this cohort of individuals with type 1 diabetes with and without diabetic polyneuropathy. We found a degeneration of nerve fibres in the epidermis and dermis and of SC processes in individuals with DPN compared with healthy control individuals and those without DPN, but found no differences when comparing those with and without pain. Additionally, a correlational analysis between dermal fibres and DSC processes with clinical measures of neuropathy indicate that they are weakly associated with neuropathy measures, thus potentially playing a role in the pathophysiology of diabetic polyneuropathy in type 1 diabetes.

### Supplementary Information

Below is the link to the electronic supplementary material.

Supplementary file1 (PDF 541 KB)

## Data Availability

Due to restrictions related to Danish law protecting patient privacy, the data used in this study can only be made available through an application to Aarhus University by contacting the corresponding author or Aarhus University (clin@au.dk).

## Abbreviations

DNF: Dermal nerve fibre
DPN: Diabetic polyneuropathy
DSC: Dermal SC
IENFD: Intraepidermal nerve fibre density
MNSI: Michigan Neuropathy Screening Instrument
MNSI-Q: MNSI questionnaire
NRS: Numeric rating scale
nSC: Nociceptive SC
nSC+nerve complex: Nociceptive SC abutting to nerve fibres
nSC−nerve complex: Nociceptive SC not abutting to nerve fibres
PNS: Peripheral nervous system
P-T1DPN: Type 1 diabetes with painful diabetic polyneuropathy (group)
ROI: Region of interest
S100B: S100 calcium-binding protein B
SC: Schwann cell
SNF: Subepidermal nerve fibre
SSCP: Subepidermal SC process
SSCS: Subepidermal SC soma
TCNS: Toronto clinical neuropathy score
T1D: Type 1 diabetes without diabetic polyneuropathy (group)
T1DPN: Type 1 diabetes with painless diabetic polyneuropathy (group)
