# Supplementary material for: Structural changes in Schwann cells and nerve fibres in type 1 diabetes: relationship with diabetic polyneuropathy
Source: Diabetologia. 2023 Sep 20;66(12):2332–45. doi: 10.1007/s00125-023-06009-z (PMC10627903; doi:10.1007/s00125-023-06009-z)
Supplement: Supplementary file 1 — Supplementary file1 (PDF 541 KB) [file 125_2023_6009_MOESM1_ESM.pdf]

Electronic supplementary material (ESM)

ESM text

Methods

SC quantifications

1) The number density of nSCs abutting to nerve fibres (nSC+nerve complex, PGP9.5$^+$/S100$^+$/SOX10$^+$/DAPI$^+$) was estimated by counting the number of nSC+nerve complexes that were within the selected ROI, $\sum Q^-$(nSC+nerve), divided by the sampling ROI_{volume} in the orthogonal max-projection confocal images. The density of nSC not abutting to nerve fibres (nSC-nerve complex, PGP9.5$^-$/S100$^+$/SOX10$^+$/DAPI$^+$) was estimated by counting the number of nSC-nerve complexes that were within the selected ROI, $\sum Q^-$(nSC-nerve), divided by the sampling ROI_{volume} in the orthogonal max-projection confocal images. The sampling ROI_{volume} was measured by multiplying the area of ROI in the orthogonally projected 2D confocal images ($\sum a(ROI_{area})$ with the thickness (21 µm) of original Z-stack images (disector height). The analysis was performed using Zeiss ZEN 3.3 (blue edition) software.

$$N_V(\text{nSC+nerve}) = \sum Q^-(\text{nSC+nerve}) / (\sum a(ROI_{area}) * \text{disector height})$$

$$N_V(\text{nSC–nerve}) = \sum Q^-(\text{ nSC–nerve}) / (\sum a(ROI_{area}) * \text{disector height})$$

2) Total dermal Schwann cell (DSC) expression level was estimated by measuring the DSC area fraction. Briefly, the DSC area fraction was estimated by measuring the area that was occupied by the total DSC ($\sum a(DSC_{area})$), divided by the total area of region of interest ($\sum a(ROI_{area})$) in the dermal area within 200 µm below epidermal-dermal border from the orthogonally projected 2D confocal images.

$$\text{DSC area fraction} = \sum a(DSC_{area}) / \sum a(ROI_{area})$$

Dermal nerve fibre area fraction

Dermal nerve fibre (DNF) expression level was estimated by measuring the DNF area fraction (the same method as measuring the DSC area fraction). Using FIJI, an individual threshold was applied to each orthogonal average-projection confocal image to exclude as much as possible of non-specific bindings from the images. DNF area fraction was estimated by measuring the DNF occupied area ($\sum a(DNF_{area})$), divided by the total area of region of interest $\sum a(ROI_{area})$, in the dermal area within 200 µm below epidermal-dermal border from the orthogonally projected 2D confocal images.

$$\text{DNF area fraction} = \sum a(\text{DNF}_{area}) / \sum a(\text{ROI}_{area})$$

Comparison between methods for dermal nerve fibre and SC quantifications

To compare the results with previously published methods, we analysed the expression of subepidermal SC (including the subepidermal SC process (SSCP) and soma (SSCS)) and subepidermal nerve fibre (SNF) using a method optimized from a published study [1]. The previously described method calculated the dermal structure density per length (not per volume as done here) and within 40 µm from the epidermal-dermal border (not 200 µm as done in DNF area fraction). Therefore, in addition to the method described above, we also quantified the solitary and total SSCS number density, SSCP density and SNF density per volume within 40 µm from the border (the modified methods), and per length within 40 µm from the border (the original methods). The details of the modified quantification methods are described below.

1) Solitary and total subepidermal SC soma (SSCS) densities (per volume) were estimated by counting the number of solitary or total SSCSs in the subepidermis within 40 µm below epidermal-dermal border, $\sum Q^-(\text{SSCS}_{solitary})$ or $\sum Q^-(\text{SSCS}_{total})$, divided by multiplying the area of selected subepidermis in the orthogonally projected 2D confocal images ($\sum a(\text{Subepidermis}_{area})$) with the thickness (21 µm) of original Z-stack images (disector height).

$$N_V(\text{SSCS}_{Solitary}) = \sum Q^- (\text{SSCS}_{solitary}) / (\sum a(\text{Subepidermis}_{area}) * \text{disector height})$$

$$N_V(\text{SSCS}_{total}) = \sum Q^- (\text{SSCS}_{total}) / (\sum a(\text{Subepidermis}_{area}) * \text{disector height})$$

2) Subepidermal Schwann cell process (SSCP) density (per volume) was estimated by counting the number of SSCPs within 40 µm below epidermal-dermal border, $\sum Q^-(\text{SSCP})$, divided by multiplying the area of selected subepidermis in the orthogonally projected 2D confocal images ($\sum a(\text{Subepidermis}_{area})$ with the thickness (21 µm) of original Z-stack images (disector height).

$$N_V(\text{SSCP}) = \sum Q^-(\text{SSCP}) / (\sum a(\text{Subepidermis}_{area}) * \text{disector height})$$

3) Subepidermal nerve fibre (SNF) density (per volume) was estimated by counting the number of subepidermal nerve fibres (SNFs) within 40 µm below epidermal-dermal border, $\sum Q^-(\text{SNF})$, divided by multiplying the area of selected subepidermis in the orthogonally projected 2D confocal images ($\sum a(\text{Subepidermis}_{area})$) with the thickness (21 µm) of original Z-stack images (disector height).

$$N_V(\text{SNF}) = \sum Q^-(\text{SNF}) / (\sum a(\text{Subepidermis}_{area}) * \text{disector height})$$

Results

Comparisons between three groups: healthy controls, T1D and DPN (painless + painful)

Post hoc comparisons showed no significant difference between participants with painless DPN and painful DPN in any of the skin biopsy results. Therefore, to investigate the relationship between the skin biopsy quantifications and diabetic polyneuropathy, we combined all participants with DPN (regardless of their pain-status) into one single DPN group. Same as the results in the four group comparisons, we also found significant differences in cutaneous SC quantifications (number density of nSC-nerve complexes, DSC area fraction, SSCP density per volume) and nerve fibre quantifications (DNF area fraction, SNF density per volume) between the three groups (all p and $p_{adj}<0.05$), but not in the number density of nSC+nerve complexes, solitary and total SSCS per volume (all $p_{adj}>0.05$; ESM Table 1).

Furthermore, post hoc comparisons in the above-mentioned five quantifications within the three study groups showed significant differences between DPN and healthy control group, as well as between DPN and T1D group (all p and $p_{adj}<0.05$); but no difference between T1D and healthy control group (all p and $p_{adj}>0.05$; ESM Table 2).

ESM Tables

ESM Table 1. Comparisons between control, T1D, and DPN (painless + painful) group

Quantification methods	Control (n=25)	T1D (n=25)	DPN (n=57)	*p* value	p_{adj} value
**Number density of nSC+nerve complex, mm^{-3}**	1598.9 (902.7;4351.3)	2773.5 (648.1;3806.6)	1036.0 (0.0;3273.8)	0.074	0.350
**Number density of nSC−nerve complex, mm^{-3}**	3684.0 (1552.2;5051.7)	3268.8 (1916.0;6234.8)	5503.8 (2594.9;8559.6)	0.034*	0.001**
**DSC area fraction, %**	0.7 (0.6;1.0)	0.7 (0.5;1.0)	0.4 (0.3;0.7)	0.002**	-
**Solitary SSCS number density, mm^{-3}**	7212.7 (4580.3;10224.3)	8155.2 (4195.6;13109.9)	6665.3 (4276.4;10523.1)	0.798	0.839
**Total SSCS number density, mm^{-}**	13782.9 (9590.0;16658.2)	15000.5 (7309.7;18192.4)	11379.9 (9083.2;15783.7)	0.487	0.920
**SSCP density, mm^{-3}**	9822.2 (7745.2;11457.4)	8846.8 (5759.8;12607.9)	4021.7 (2088.0;6926.1)	<0.001***	<0.001***
**DNF area fraction, %**	0.9 (0.8;1.2)	0.8 (0.6;1.1)	0.6 (0.5;0.8)	<0.001***	-
**SNF density, mm^{-3}**	11077.9 (8073.7;12644.4)	10823.9 (6355.5;12833.6)	4747.3 (2453.4;8129.7)	<0.001***	<0.001***

All continuous variables were non-normally distributed and were presented as median (IQR). After square root transformation, variables were analyzed using both ANOVA (*p* value) and ANCOVA (p_{adj}). value, adjusting for age, sex and HbA1c). Variables that could not be transformed were analyzed by Kruskal-Wallis H test (non-adjusted *p* value).

**p* values < 0.05; ***p* values < 0.01; and ****p* values < 0.001.

ESM Table 2. Post-hoc comparisons for skin biopsy quantifications between control, T1D, and DPN (painless + painful) group

Quantifications	Groups	Mean difference	95% CI (lower)	95% CI (upper)	*p* value	p_{adj} value
**Number density of nSC−nerve complexes, mm^{-3}**	T1D vs Control	464.7	-25.6	2320.9	-	0.154
	DPN vs Control	1820.7	199.6	5071.4	-	0.001**
	DPN vs T1D	445.8	0.2	1741.7	-	0.043*
**DSC area fraction, %**	T1D vs Control	-	-	-	1.000	-
	DPN vs Control	-	-	-	0.003**	-
	DPN vs T1D	-	-	-	0.011*	-
**SSCP density, mm^{-3}**	T1D vs Control	-26.1	-859,9	365.3	-	1.000
	DPN vs Control	-1220.5	-3709.3	-80.4	-	0.004**
	DPN vs T1D	-889.8	-2361.1	-122.5	-	0.001**
**SNF density, mm^{-3}**	T1D vs Control	-45.1	-1006.2	334.6	-	1.000
	DPN vs Control	-1440.7	-4195.3	-124.1	-	0.002**
	T1DPN vs T1D	-976.1	-2561.8	-140.9	-	<0.001***
**DNF area fraction, %**	T1D vs Control	-	-	-	0.520	-
	DPN vs Control	-	-	-	<0.001***	-
	DPN vs T1D	-	-	-	0.018*	-

Square root transformed (parametric) data are presented as adjusted *p* value and mean difference with 95% confidence interval analyzed by pairwise comparisons *t* tests adjusting for age, sex and HbA1c, and non-parametric data are presented as non-confounder adjusted *p* value by using Dunn’s test.

**p* values < 0.05; ***p* values < 0.01; and ****p* values < 0.001.

ESM Figures

ESM Fig. 1

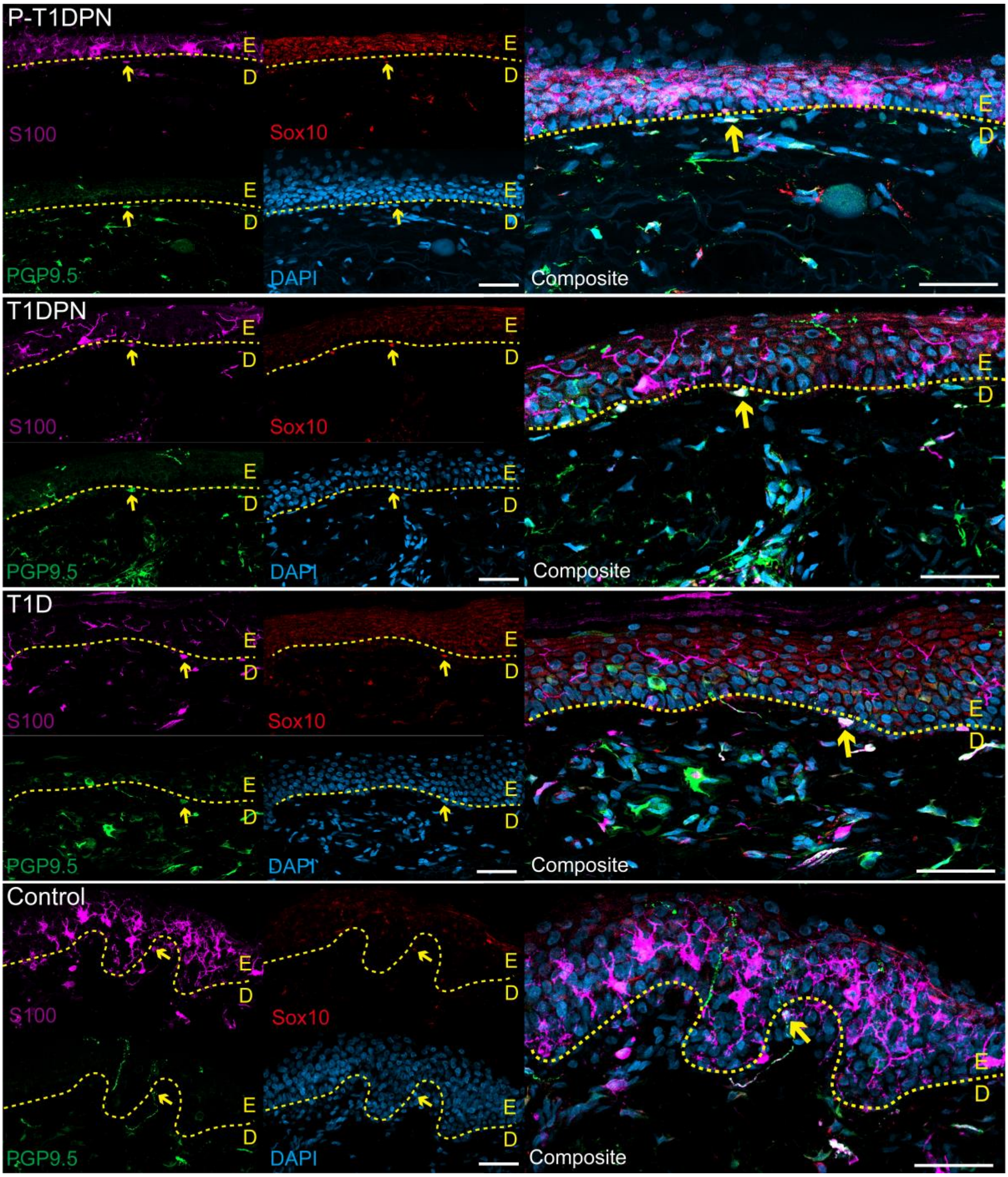

Representative immunofluorescent staining of SCs and peripheral nerve fibres in each group. T1D: type 1 diabetes without diabetic polyneuropathy; T1DPN: type 1 diabetes with painless diabetic polyneuropathy; P-T1DPN: type 1 diabetes with painful diabetic polyneuropathy. Scale bar, 50 μm in all images. Arrows: nSC+nerve complex, yellow dotted lines: epidermal-dermal border. E: epidermal; D: dermal.

Reference

1. Özdağ Acarlı AN, Klein T, Egenolf N,Sommer C (2022) Subepidermal Schwann cell counts correlate with skin innervation - an exploratory study. 65(4): 471-479. 10.1002/mus.27496
